# Few-Shot Learning for Plant-Disease Recognition in the Frequency Domain

**DOI:** 10.3390/plants11212814

**Published:** 2022-10-22

**Authors:** Hong Lin, Rita Tse, Su-Kit Tang, Zhenping Qiang, Giovanni Pau

**Affiliations:** 1 Faculty of Applied Sciences, Macao Polytechnic University, Macao SAR 999078, China; 2 College of Big Data and Intelligent Engineering, Southwest Forestry University, Kunming 650224, China; 3 Department of Computer Science and Engineering, University of Bologna, 40126 Bologna, Italy; 4 Samueli Computer Science Department, University of California, Los Angeles, CA 90095, USA

**Keywords:** few-shot learning, plant disease recognition, frequency domain, Gaussian-like calibration, discrete cosine transform, power transform

## Abstract

Few-shot learning (FSL) is suitable for plant-disease recognition due to the shortage of data. However, the limitations of feature representation and the demanding generalization requirements are still pressing issues that need to be addressed. The recent studies reveal that the frequency representation contains rich patterns for image understanding. Given that most existing studies based on image classification have been conducted in the spatial domain, we introduce frequency representation into the FSL paradigm for plant-disease recognition. A discrete cosine transform module is designed for converting RGB color images to the frequency domain, and a learning-based frequency selection method is proposed to select informative frequencies. As a post-processing of feature vectors, a Gaussian-like calibration module is proposed to improve the generalization by aligning a skewed distribution with a Gaussian-like distribution. The two modules can be independent components ported to other networks. Extensive experiments are carried out to explore the configurations of the two modules. Our results show that the performance is much better in the frequency domain than in the spatial domain, and the Gaussian-like calibrator further improves the performance. The disease identification of the same plant and the cross-domain problem, which are critical to bring FSL to agricultural industry, are the research directions in the future.

## 1. Introduction

Plant disease is a significant factor that reduces the quality and harvest in the crop production [1]. In recent years, deep learning in computer vision has obtained excellent achievements. In agriculture industry, image-based deep learning methods for auto-diagnosing have raised great concerns due to their accessible and economical characteristics [2]. Once the technology is applied, it will benefit farmers and the agriculture industry greatly. However, the data shortage has been an obstacle to taking advantage of deep-learning methods [2]. As we know, deep-learning methods always rely on large-scale data. However, the existing data resources are very limited in plant-disease recognition. Meanwhile, creating large-scale datasets of plant-disease images is an exhausting task that needs experts’ involvement. Some approaches have been proposed to alleviate the data shortage, including data augmentation, transfer learning, few-shot learning (FSL), etc.

FSL has been proposed in recent years, which can generalize to novel categories with the support of few samples. It has been effective at alleviating the hard requirement of data. One branch of FSL is the metric-based method [3]. Its principle is that the features of samples belonging to the same category are close to each other, and the features of samples belonging to different categories are far from each other. The earliest representative work was the Siamese network, which is trained with a positive sample pair or negative sample pair [4]. Then, some classical networks were proposed successively. Matching networks borrow the concept “seq2seq+attention” to train an end-to-end nearest neighbor classifier [5]. The prototypical network learns to match the proto-center of the class in semantic space through few samples [6]. The relational network concatenates the feature vectors of the support samples with those of the query samples to discover the relationship of classes [7]. CoveMNet extracts the second-order statistic information of each category by an embedding local covariance to measure the consistency of the query sample with the novel classes [8]. In the meta-baseline method, which is based on a naive metric-based concept, the centroid of category is the mean vector of the support samples. It measures the distances from the query sample to the centroids of novel categories [9].

Recently, FSL has started to be used in the research of plant-disease recognition. The Siamese network and the dataset PlantVillage (PV) were used in [10,11]. A generative model using a conditional adversarial auto-encoder was proposed for identification of citrus aurantium L. diseases [12]. A semi-supervised FSL approach was proposed and PV was used in [13]. A matching network was also used to test cross-domain performance by mixing pest data [14]. Baseline [15], Baseline++ [15] and DAML [16] were compared on PV and the Coffee Leaf Dataset in [17]. In addition, some methods, such as multi-scale feature fusion, attention, transformer etc. were also used to improve the performance of FSL [18,19,20,21]. These existing works have been tried from various perspectives and have made important progresses; however, they are all focused on the spatial domain.

In this work, we intended to take advantage of FSL to deal with the shortage of data and improve the identification accuracy. While FSL has many advantages in dealing with data limitations, it still has two common challenging issues: (1) The limited features extracted from a few samples are poorly representative of the categories. (2) The requirement of generalization ability is demanding. Thus, improving the representation of features and the generalization of the model are the urgent problems in FSL research. The conventional studies of classification tasks are mainly focused on the spatial domain [22]. In this work, we aimed to tackle these problems from another perspective: shifting the conventional spatial domain to the frequency domain to enhance the feature representation. Additionally, we adopted the Gaussian-like calibration (GC) to improve the generalization ability of the model.

In computer vision, most of the existing studies of classification [22], segmentation [23], object detection [24] etc., are focused on the spatial domain. The frequency representation is mainly used for image compression, such as JPEG encoding [25,26]. The underlying rationale is that the human visual system has unequal sensitivity to different frequency components. Hence, for compression reasons, the unimportant frequencies can be abandoned without much degradation of image quality. Meanwhile, it has been validated that frequency representations can be fed to the convolutional neural network (CNN) to extract features. Coincidentally, as with the human visual system, the CNN model is more sensitive to low-frequency channels than high-frequency channels [27]. In 2020, the use of frequency representations was shown to achieve impressive performance in classification task using various CNNs as backbone networks [28]. In 2021, a method of integrating spatial representations and frequency representations together was proposed, and the experiments show that the performance in the frequency domain is higher than in the spatial domain [29]. The existing works demonstrate that a compressed frequency representation still contains rich patterns for image understanding tasks [28]. Inspired by these works, we take advantage of the frequency representations of features in the FSL classification task and use the discrete cosine transform (DCT) as the transformer to transfer from the spatial domain to the frequency domain.

The DCT is one of the most efficient transform coding schemes and was introduced in [30]. It is an orthogonal, separable and real transform which translates the image information from the spatial domain to the frequency domain, to be represented in a more compact form [31,32]. In JPEG encoding, the DCT is used to improve the efficiency because not all frequency channels are equally important for the feature representation [33]. According to the human visual mechanism, the low frequencies are kept and the high frequencies are abandoned without much accuracy loss [28].

To improve the generalization of model, we discuss the distribution of the features extracted from CNN and calibrate the skewed distribution. Although the mainstream works using deep-learning neural networks involve feature extraction, few have explored the distributions of the features. The features extracted from the encoder networks (e.g., CNN) are hypothesized to be aligned with a particular distribution. Generally, one assumes that the samples of the same category are aligned with a certain distribution, and the samples from the same dataset are aligned with the approximate distributions. However, these assumptions are rarely experimentally discussed and demonstrated. The main problem is that the distributions of the features extracted using a backbone architecture are complex. In fact, it is very likely that they are not of a Gaussian distribution or other distribution. Unfortunately, the problem is enlarged in FSL, because it is a method that requires generalization to new categories. The big differences in the feature distributions in training and testing increase the difficulty of generalization. In particular, the distance-metric methods are sensitive to the distribution of features. If the vectors are aligned with the same distribution (e.g., Gaussian-like distribution) before measuring the distance between them, then these aligned features will help improve the generalization of model. The significance of distribution calibration in FSL is introduced in [34].

In statistics, the power transform (PT) denotes a family of functions applied to create a monotonic transformation of data using power functions. It is a data transformation technique used to stabilize variance and make the data more Gaussian-like [35]. The PT was used in the pre-processing of feature vectors before they were fed into the classifier in [36,37]. In this work, we designed a GC module to calibrate a skewed feature distribution.

In the pipeline of the network architecture in this work, there is a DCT module to transform 2D color images from the spatial domain to the frequency domain, and a GC module for post-processing after feature extraction and before distance measurement. The main contributions of this work are summarized as follows:

(1) This is the first work to introduce plant-disease recognition in the frequency domain.
(2) We adopted DCT to transform data to the frequency domain. For the DCT module, we propose a learning-based frequency selection method to improve the adaptability to different datasets or data settings. In addition, we designed a GC module to align the skewed distributions of feature vectors to a Gaussian-like distribution. The two modules can be flexibly ported to other methods or networks.
(3) We conduct extensive experiments to explore the DCT module and GC module. Compared with the related methods, the accuracy our method is state-of-the-art.

The rest of this paper is organized as follows: Section 2 introduces all experiments and analyses results; Section 3 is the discussion part; Section 4 introduces the hardware configurations, data settings and our methods including the problem definition, framework, DCT module, Gaussian-like calibrator and distance measurement; the paper is concluded in Section 5.

## 2. Experiments and Results

We carried out 47 comparison experiments and ablation experiments to illustrate our method in detail. The details of experiments and results are illustrated and analyzed as below. All results are average accuracy (%) of 10 epochs in the test. The N-way was set as 5. The ResNet12 was chosen as the feature extraction backbone network. We set up data pre-processing before feeding it into the feature extractor. In the spatial domain, the pre-processing includes:

- RandomResizedCrop;
- RandomHorizontalFlip;
- Normalize.

In the frequency domain, the data-processing includes:

- Resize(int(filter_size*image_size*1.15)) (before DCT);
- CenterCrop(filter_size*image_size) (before DCT);
- Normalize (after DCT).

Resize was used to enlarge the image_size by up to filter_size*1.15 times first. CenterCrop is used for getting the center part of the resized image. As mentioned in Section 4.2.3, the size of each channel is image_size/filter_size after transformation. Resize is used for keeping the size of each channel the same as the image_size after DCT.

### 2.1. Comparison and Ablation Study

For showing the effectiveness of frequency representation and GC module, we conducted experiments with three data splits to compare the spatial domain, spatial domain with GC, frequency domain and frequency domain with GC. The experiments on the spatial domain were without the DCT module but feeding the RGB image into encoder directly. The results are listed in Table 1 and the three data settings are introduced in Section 4.1. e1–e4 were conducted with data setting-1. e5–e8 were conducted with data setting-2. e9–e12 were conducted with data setting-3. In each group, the lowest accuracy was for the spatial domain. The accuracy in the frequency domain was much higher compared to that in the spatial domain. The GC can be added in either the spatial domain pipeline or the frequency domain pipeline, and obviously improves the accuracy in both domains. The method of using DCT and GC resulted in the best accuracy in all three data settings. In the case of 1-shot, accuracy was improved by more than 7% in the three data settings.

### 2.2. Channel Selection

As introduced in Section 4.2.3, the more impactful frequency channels will be selected from Y,Cb and Cr frequency cubes to be the final frequency representation. There are two ways of selecting frequency channels: fixed channel selection and learning channel selection. The fixed selection method is based on some principles and experiences. Generally, the more impactful channels are in low-frequency band. The upper part of the minor diagonal (the left-bottom to right-top one) is considered as the low frequency part, and the lower part is considered as the high frequency part. However, at least half of the frequency channels (including the diagonal) are still in the low-frequency band. Such a crude selection is not beneficial for computational efficiency. Alternatively, some works have proposed heatmaps of some popular datasets (e.g., Imagenet) [28]. Actually, different datasets have different combinations of informative frequency channels, which was verified in [28]. The coarse or empirical selection can not adapt to different datasets very well. Thus, it is not reasonable to use static frequency combinations for different datasets. We use two experiments to illustrate the effects of different frequency combinations. As shown in Table 2, e13 used the frequency combination according to the heat map of ImageNet [28] and e7 used the learning frequency combination of data setting-2 proposed in this work. The results show that even such a large-scale dataset cannot generalize to different datasets very well. Using the corresponding frequencies for specific datasets is necessary.

**Figure 1:**
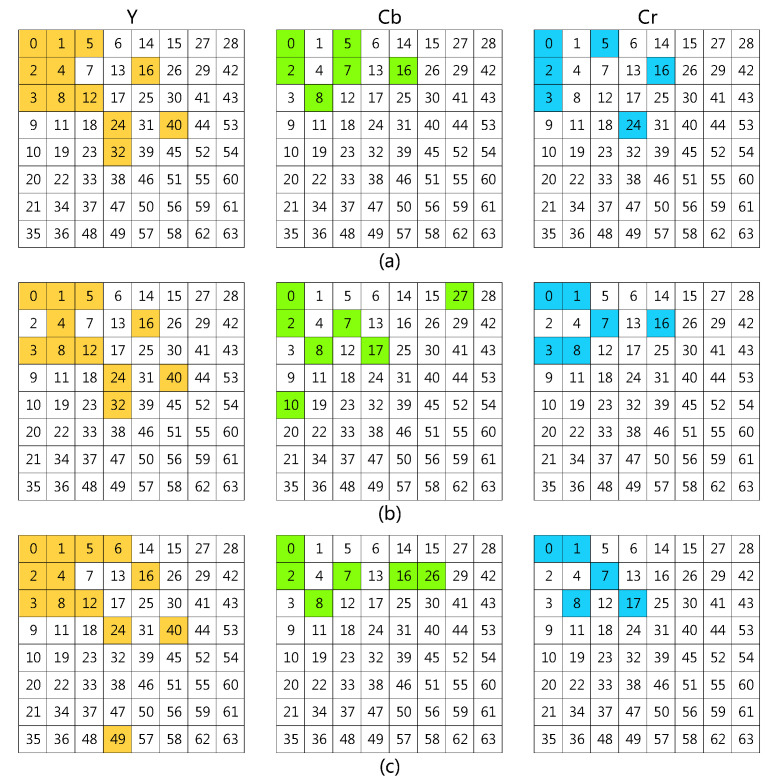
(**a**) The frequency selection of setting-1. (**b**) The frequency selection of setting-2; (**c**) The frequency selection of setting-3. The colored grids are the selected frequencies. Yellow grids are frequencies selected from *Y*, green grids are frequencies selected from Cb and blue grids are frequencies selected from Cr. The number of the selected grids was 24. e3 and e4 used the frequencies listed in (**a**). e7 and e8 used the frequencies listed in (**b**). e11 and e12 used the frequencies listed in (**c**).

In this work, we propose to use a learning method to select the informative frequency channels. In [38], the Squeeze-and-Excitation (SE) attention module was used to learn the attention of channels. In our method, we adopt the SE channel attention module to learn the significance of each frequency channel. Taking the standard filter-size 8×8 as an example, the full frequencies are 192 channels. The SE module calculates 192 values as weights of the 192 frequencies. Top-N channels are selected according to the weights. For the three data settings, from the 192 frequencies obtained with the 8×8 filter, the top-24 frequencies are selected using the learning method, which is shown in Figure 1.

We found that most of the selected channels are indeed in the low-frequency band. However, the layout of these frequencies does not follow a regular triangular or rectangular arrangement, but is asymmetrical [28]. Even though all three data settings were for the same dataset, different categories and samples still exhibit different frequency sensitivities. Thus, for different datasets or data settings, the learning-based selection method helps to obtain the optimal combination of frequencies.

### 2.3. The Effect of Top-N

In this section, we explore the effect of the number of selected frequencies. In Table 3, we compare the top-N frequencies: top-8, top-16, top-24, top-32, top-48 and top-64. This group of experiments was performed with data setting-2. It showed that there are more channels from *Y* than Cb and Cr in the *N* selected frequencies, which means that *Y* contains more informative features. It verifies the theory of image compression again. The best accuracy of 1-shot was with the top-24, and the best accuracies of 5-shot and 10-shot were with the top-64. The accuracy with the top-24 did not degrade too much compared with that of the top-64, but the communication bandwidth was reduced greatly. In this work, we choose the top-24 as the optimal top-N to achieve a balance of accuracy and efficiency. The results for e19 using only the frequencies of the *Y* channel decrease sharply, suggesting that some frequencies of Cb and Cr are also important for this classification task.

### 2.4. The Effect of High Frequency

From the previous experiments, the most informative frequencies are in low-frequency bands. Hence, we conducted another group of experiments to explore the effect of selection range. Three ranges using the 8×8 filter were tested: the full 192 frequencies; the 108 frequencies of the upper triangle, including the minor diagonal; and the 84 frequencies of the upper triangle without a minor diagonal. The results of e7, e20 and e21 using the top-24 channels that were, respectively, selected from the three ranges are shown in Table 4. The best performance was the top-24 of the full 192 frequencies, which indicates that even if the more influential frequencies are in the lower-frequency bands, it is not an absolute rule. Some high frequencies also have important contributions to our task, such as the number 40 channel in *Y*.

### 2.5. The Effect of Filter-Size

In this subsection, we explore the effect of filter-size. As analyzed in Section 4.2.3, $S_{DCT}\times S_{DCT}$ is the filter-size of the DCT. The size can be a standard setting of 8x8 pixels or other different integer sizes between 4×4 and 32×32 pixels [39]. The number $S_{DCT}\times S_{DCT}$ represents the frequency granularity. A small number means a coarse frequency separation, and large number means a fine frequency separation. For example, the 8×8 filter means *Y*, Cb and Cr channels are transferred to 64 frequencies, and the full frequencies is 192. The 16×16 filter transforms *Y*, Cb and Cr to 256 frequencies, and the frequencies number 768. We compare three filter-sizes, 4×4, 8×8 and 16×16, and the results are shown in Table 5. e22–e24 used a 4×4 filter, e7, e25 and e26 usd ae 8×8 filter, and e27–e29 used a 16×16 filter. The selected top-N frequencies follow the ratios: 1/8, 1/4 and 1. The best result used the standard 8×8 filter with the top-24 frequencies.

### 2.6. Ablation Experiment of the GC Module

In the GC module, in addition to the core part of PT, we have vertical sliding, normalization and centralization. The vertical sliding is a necessary step to solve to negative-value problem. We carried out ablation experiments to show the effects of the other two components, as shown in Table 6. Compared to e30, e31 and e32 show the contributions of the normalization and the centralization, respectively. In e33, the normalization was performed before PT. The best setting of GC module was PT+normalization+centralization, that is, the configuration of e8.

**Figure 2:**
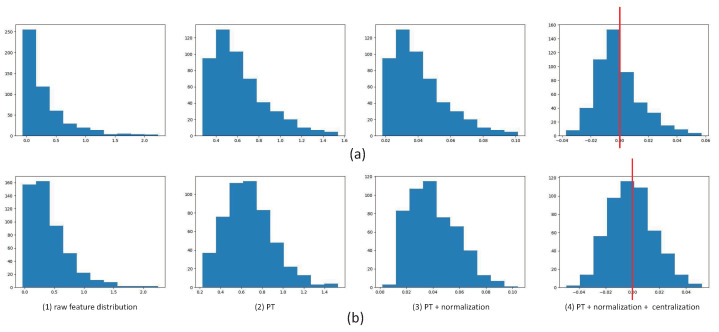
The histograms of each step in the GC module. The red line highlights the symmetry of the histogram when μ=0. (**a**) A sample embedded in the spatial domain. (**b**) The same sample embedded in the frequency domain. The first column is the raw feature distribution, the second column is after PT, the third column is after PT + normalization, the fourth column is after PT + normalization + centralization.

In order to see the effect of the Gaussian-like calibrator, we use a histogram to show the distribution of the feature vectors. In Figure 2, the first row shows a sample embedded in the spatial domain and the second row shows the same sample embedded in frequency domain. The first column shows the raw feature distribution; the second column shows the distribution after calibration; the third column shows the distribution after PT and normalization; and the last column shows the distribution after PT, normalization and centralization. Obviously, PT adjusts the right-skewed distribution to a Gaussian-like distribution. The centralization makes the distribution more standardized and symmetrical. The distribution in the frequency domain is more like a Gaussian distribution than that in the spatial domain, which is one of the reasons why the performance in the frequency domain is better than that in the spatial domain.

### 2.7. Setting of β

**Figure 3:**
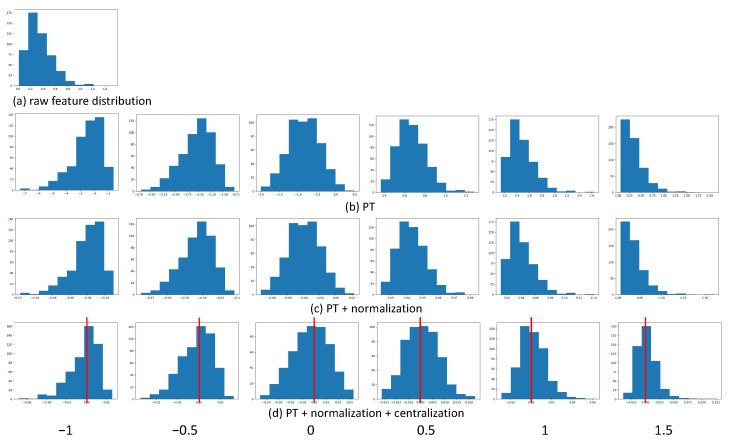
The histograms of different β of each step in the GC module. The red line highlights the symmetry of the histogram when μ=0. (**a**) The raw feature distribution. (**b**) After PT. (**c**) After PT + normalization. (**d**) After PT + normalization + centralization.

According to the definition of PT, the degree of calibration is decided by the hyper-parameter β. β=1 leads no effect, and decreasing β can phase out the right-skewed distribution [36]. We carried out a group of experiments to observe the effects of different β, as shown in Table 7. As observed, most of our data are right-skewed. The value of β starts at 1.5 and decreases gradually. The histograms of the feature distributions are shown in Figure 3. Figure 3a is the raw feature distribution which is a right-skewed example, Figure 3b is the distributions after PT, Figure 3c is the distribution after PT+normalization and Figure 3d shows the distribution after PT+normalization+centralization. It is clear that after centralization, β=0.5 leads to the closest normal distribution calibration, which is consistent with the results of e8. The first column and the second column are left-skewed distributions because the β is negative. The optimal setting of β is 0.5 or 0. The β in e35 is 1, which means no effect of PT. However, the results of e35 are better than e7 because the normalization and centralization still work and have positive effects. The degree of normalization of the distributions and the results shows a positive correlation. The closer the morphology of the distribution to the normal distribution, the higher the accuracy. This group of experiments demonstrated that the distribution calibration is necessary.

### 2.8. Integrating Spatial and Frequency Representation

**Figure 4:**
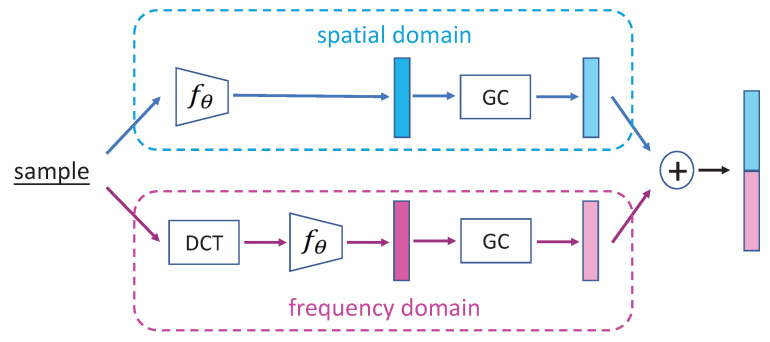
The network architecture of integrating spatial and frequency representations.

We carried out another experiment of integrating the spatial representation and frequency representation together. The framework is shown in Figure 4. A sample was fed into the spatial branch and one into the frequency branch, and two feature vectors were generated. Then, the two feature vectors were concatenated as the representation of the sample. The results are shown in Table 8. It is unexpected that the results of integrating method are not better than the results in the frequency domain only. The reason is discussed in Section 3.

### 2.9. Efficiency Analysis

**Figure 5:**
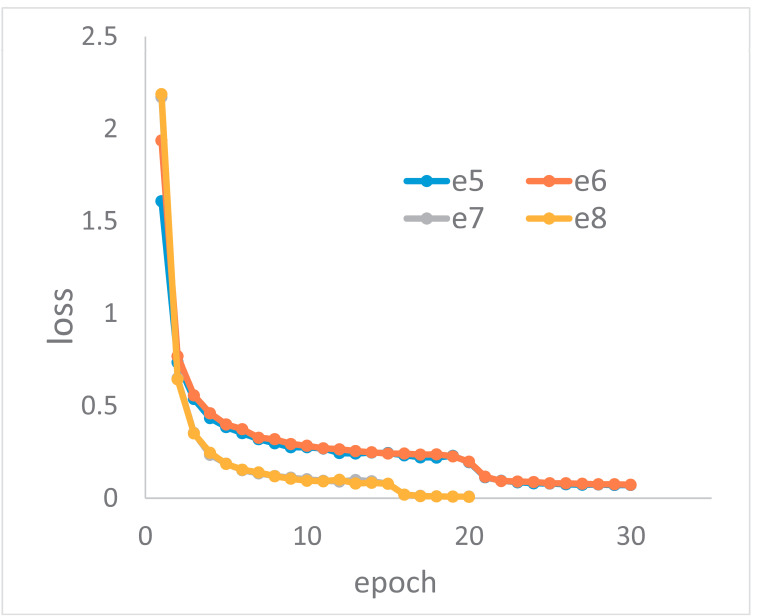
The loss in the spatial domain and frequency domain in the pre-training stage.

In our experiments, we found that the convergence speed of backbone CNNs in spatial domain is very different from in the frequency domain. The four loss curves of e5, e6, e7 and e8 in the pre-training stage are shown in Figure 5. e5 and e6 are in the spatial domain, and e7 and e8 are in the frequency domain. The network was convergent within 20 epochs, and the loss declined to around 0.008 in frequency domain. In the spatial domain, the network was convergent within 30 epochs, and the loss declined to around 0.07. This means that CNNs are very efficient at extracting features from frequency representations.

### 2.10. Comparison with Related Works

In order to show the superiority of our method, we conducted many experiments to compare our method with the recent related ones. We used other data settings of PV: 32 categories for training and the remaining 6 categories (apple 4 classes, blueberry healthy, cherry healthy) for testing. We compared it with the work of [10,13,40] (e40, e41, e42, e43). We also used three other data settings to compare with [13] (e9, e10, e11, e12; e5, e6, e7, e8; e44, e45, e46, e47). In order to be comparable, we executed the experiments with the same data settings as their work. The results are shown in Table 9. The related frequency combinations of each data setting are listed in this table too.

## 3. Discussion

### 3.1. Motivation, Works and Contributions

FSL is currently in the rapid development phase of theoretical research and has just started to be used in smart agriculture. Although FSL is very suitable for plant disease identification and has attracted active research in recent years, its accuracy is not so confident when applying this technique. These reasons motivated us to further improve the accuracy of plant disease identification. After an exhaustive literature review, we found that the existing studies in this field are all in spatial domain. Therefore, we intended to explore FSL from a new perspective, i.e., in the frequency domain.

Inspired by the image compression technique, we proposed an FSL method in the frequency domain for plant-disease recognition. In the framework, the RGB image is transferred to the frequency domain by the DCT module. Then, the frequency representation is fed to the CNN for feature extraction. In order to align the feature vectors with a Gaussian-like distribution, we designed the GC module to calibrate the skewed distribution. Through extensive experiments, the effectiveness of the two modules were explicitly illustrated, and the configurations of DCT module and GC module were analyzed. No matter the data settings, and in comparison to related studies, the superiority of our method indicates that our method has good universality.

Our work is the first work on FSL for plant-disease recognition in the frequency domain. In the DCT module, we proposed a learning-based frequency selection method for improving the adaptability to different datasets or data settings. We designed a GC module to align these complex distributions with a Gaussian-like distribution, which has positive effects in both the spatial domain and the frequency domain. The two modules can be used independently from our pipeline as plug-ins for other methods or networks. In particular, they can be easily transplanted to the popular CNNs, suggesting that these two modules have a wide range of application scenarios. Comparing with the related works, our method is state-of-the-art.

### 3.2. Findings

1. The performance was improved in the frequency domain compared to in the spatial domain, which indicates that better features can be obtained from frequency representation. In other words, CNNs can extract more critical features from a frequency representation. Another proof demonstrates this point, which is that the convergent speed was much faster for the frequency domain than for the spatial domain, and the loss was smaller as well. This important finding suggests that the frequency representation in the frequency domain can be attempted not only for classification tasks in FSL, but also for other classification tasks, and even for any other task that uses a CNN to extract features.
2. In this work, we used cosine similarity to calculate the distance, and the GC module has shown prominent improvements in both the frequency domain and the spatial domain. Our method illustrates the use of Gaussian-like calibration in a CNN pipeline. In fact, the GC module can be used in a broader range of machine learning. Several researches have shown that not only cosine similarity, but also other distance-based methods, such as KNN, K-means and the SVM classifier, are worth using on PT to improve performance.
3. We have demonstrated that channel selection in DCT is critical to determine the performance. Different frequency combinations lead to very different results. As the frequencies are considered as channels, the channel attention can be used to calculate the weights of different frequencies. We provided an idea for frequency selection. In this work, the SE module was used. Actually, it can be replaced with other channel attention modules, such as the channel attention module in the CABM, thereby providing great flexibly [42].
4. In principle, performance is always lifted when adding more information. However, the experiment of combining the spatial and frequency representations together did not show an improvement, but a detriment. According to our analysis, the spatial representation and the frequency representation are two ways of representing the same entity, but the substance of the content is the same. In the whole pipeline, the performance is also decided by the parsing ability of the backbone network to a certain representation of sample. The combination of the spatial representation and frequency representation does not enrich the features or generate extra useful information. Conversely, redundant representation become the interference factor.
5. In recent years, the architectures of networks have gone deeper and deeper. However, it does not mean that deeper networks always outperform shallower networks. First, FSL is a kind of learning task with limited data-scale. For a deeper network, it always has a large number of parameters that need to be updated. Under the data-limitation condition, too deep a network could result in insufficient updating of parameters in back-propagation due to the overly long back-propagation path. In parameter updating, shallower networks are more flexible, and the deeper networks are bulky. In addition, our specific task identified diseases while relying on the colors, shapes and textures of lesions. These simpler and more basic features are learnt in shallower layers. Hence, the very deep networks are not helpful. In short, the size of network should match the specific task and data resources.

### 3.3. Limitations and Future Works

1. Multi-disease cases are not involved in this work. In short, the samples of PV were taken under controlled conditions (laboratory settings). These settings make the samples relatively simple and differ significantly from those obtained under in-field conditions. That is the reason many researches have already achieved high accuracy by using deep-learning CNNs on PV [43]. Since the aim of this work was to explore methods in the frequency domain and we did not want the content of this paper to be scattered, we only used PV in our experiments. Therefore, the multi-disease cases were not taken into account in this work. In fact, once infected by the first disease, the plant is vulnerable to other diseases, as the immune system is attacked and becomes weak [44]. In real field conditions, it is relatively common for multiple diseases to occur in one plant. However, the combinations of different diseases are too many to collect sufficient samples for each category from classification perspective (e.g., three diseases of a species generate seven categories). Hence, if using classification to solve this problem, requirements of data are difficult to meet. We recommend using semantic segmentation to solve this problem.
2. As shown in Section 4.1, the images of PV have simple backgrounds. Complex background cases were not considered in this work. In the application, we could not predict the scenario in the test. Images may be taken from fields with complex backgrounds. In order to improve the accuracy, it is necessary to perform pre-processing to reduce the influence of the background, which involves the research direction of object detection (e.g., leaves, fruits or lesions detection). In future work, research on plant disease identification should shift to real field environments. The treatment of complex environments is important to advance the technology into practical applications.
3. In this work, we set up three data settings to mimic different application scenarios. The results of setting-1 and setting-2 are far superior to those of setting-3. setting-1 is the easiest setting because the features of different plants are very distinguishable. Setting-1 covers 10 plants, and setting-2 covers 3 plants. The results of setting-2 got close to those of the setting-1, which indicates that the model not only identified the plants but also the diseases. The performance of setting-3 with 10 test categories belonging to tomato dropped. This kind of setting is more meaningful to farmers but is a difficult task. To some extent, this is the purest form of disease identification, without any notion of species. It is sub-class classification (fine-grained vision classification), which is another research topic for computer vision. It needs more finely distinguishable features, which like humans, distinguish two similar objects. The lesion detection can help the networks focusing on the fine-grained features and improve the sub-class classification task.
4. Cross-domain research is a hot topic in FSL recently, which is defined as training with one dataset and generalizing to another dataset in the test. Our work does not refer to this topic because only one dataset was used. Further mining the benefits of feature distribution may solve the cross-domain problem. In PV, although there are differences between categories, the distributions of the samples are similar because these samples were collected under the same conditions. The data from different datasets would lead to more complex distributions. The feature distributions of different datasets and the distribution calibration of the different domains are worth studying.
5. Another focus is the fine research of the frequency channels. In this work, although we evaluated the weights of each channel, we did not do further research of these channels. In future work, some visualization works and fine studies of each channel could help to better understand the frequency representation.
6. Even though we demonstrated that CNNs can extract better features from frequency representations, networks that are more suitable for frequency representations are worth being investigated.

## 4. Materials and Methods

### 4.1. Materials

The configuration of hardware used in this work was: graphics: Tesla V100-DGXS-32 GB; video memory: 32G×4; processor: Intel(R) Xeon(R) CPU E5-2698 v4 @ 2.20GHz; operating system: Ubuntu 18.04.6 LTS.

**Figure 6:**
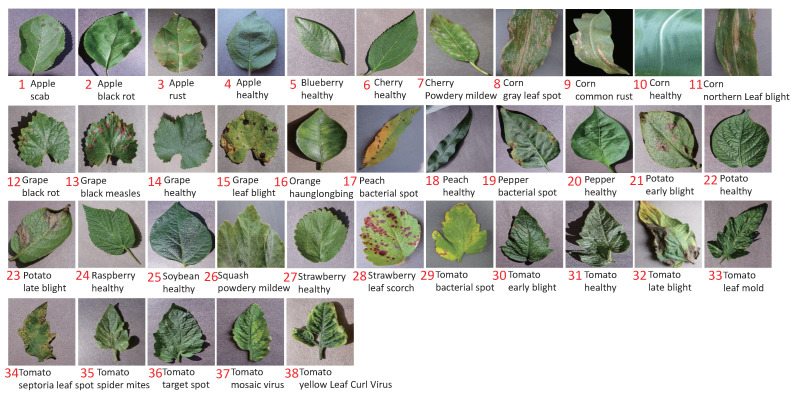
The samples of 38 categories in PV.

For the data, we used PV [45] as our experiment materials, which was released in 2015 by Pennsylvania State University. It is the most frequently used and comprehensive dataset in academic research up to now in plant-disease recognition. It includes 50,403 images which cover over 14 crop species and 38 categories. As shown in Figure 6, each category is given an ID. The characteristics of PV are: (1) a single leaf is included in per image; (2) a single disease occurs per leaf; (3) the backgrounds of the images were unified by using a clean board; (4) the illumination was controlled when taking photos.

According to the requirement of data setting in FSL, data should be split into two parts without intersection: source set and target set. The source set is used for training, and the target set is used for testing. We set up three data settings, as illustrated in Table 10: (1) setting-1: 10 categories covering 10 species were selected as the target set, and the remaining 28 categories were used as the source set; (2) setting-2: 10 categories covering 3 species were selected as the target set, and the remaining 28 categories were used as the source set; (3) setting-3: 10 categories belonging to tomato only were selected as the target set, and the remaining 28 categories were used as the source set.

The three settings represent three difficulties: setting-1 is the easiest task because it consists of 10 different species. The features of different species are more distinguishable. setting-2 is the middle-difficulty task due to it covering 3 species. The most difficult setting is setting-3, because all samples only belong to tomato and the samples are similar to each other. As the number of samples in PV is unbalanced, we used the data after augmentation and selected 1000 images per category to maintain balance.

### 4.2. Method

#### 4.2.1. Problem Definition

In the FSL paradigm, given two labeled sets with categories $C_{train}$ and $C_{novel}$, $C_{train}$ is used in training and $C_{novel}$ is used in testing. The two sets are exclusive, $C_{train}\cap C_{novel}=⌀$, which means that categories used in the test are not seen during training. Data are formulated to tasks, and each task *T* is made up of a support set *S* and a query set *Q*. The definition of *T* is formalized as:

(1) $$T=(S,Q)=\left((N-\mathrm{Way},K-\mathrm{Shot}),(N-\mathrm{Way},W_{-\mathrm{query}\;\mathrm{samples}})\right)$$

The sample of *S* is denoted by $\left(x_{s},y_{s}\right)$, and the sample of *Q* is denoted by $\left(x_{q},y_{q}\right)$, which are (image,label) pairs. In training, the label $y_{q}$ is used for calculating loss, which is supervised learning. An N-way,K-shot task indicates that the *S* contains *N* categories with *K* samples in each category; the *Q* contains the same *N* categories with *W* samples in each category. The goal is to classify the N×W unlabelled samples of Q into *N* categories.

#### 4.2.2. Framework

**Figure 7:**
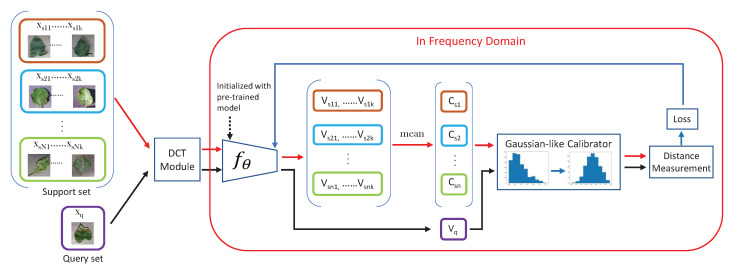
The network’s architecture.

The pipeline of our work contains four components: the DCT module, $f_{\theta }$, GC module and distance measurement module, which is illustrated in Figure 7. The original RGB image is input into the DCT module first to be transferred to the frequency domain. The output of the DCT module is a combination of different frequencies, which is then fed to $f_{\theta }$ for feature extraction. $f_{\theta }$ can be any CNN, such as Resnet or Densenet, which is initialized with the pre-trained model. The pre-trained model is trained in the pre-training stage with source data or other large-scale datasets (e.g., Imagenet) in an image-wise format, which is considered as prior knowledge. Then, in the meta-learning stage, $f_{\theta }$ is initialized by the pre-trained model, and the classifier is a fully connected layer in the pre-training stage, which is used in a distance measurement module. To these feature vectors, before measuring the distances, a Gaussian-like calibration module is arranged for calibrating the skewed features. At the end, the aligned feature vectors are used to measure distances and determine the classification results.

FSL is a task-driven paradigm where data are formulated as tasks. Given a N-way,K-shot task, all samples of *S* are transferred to frequency representations by DCT, which are then embedded into feature space by $f_{\theta }$ and become feature vectors. A mean vector of *K* feature vectors of category $C_{i}$ is calculated as the centroid of $C_{i}$, which is considered as the representative of category $C_{i}$:

(2) $$C_{i}=\frac{1}{K}\sum_{j\in K}f_{\theta }\left(d\left(x_{sij}\right)\right)\;\left(i\in N\right)$$

where $x_{sij}$ denotes the *j*th sample of the *i*th category in *S*, *d* is the DCT, *K* is the number of shots in each category and $C_{i}$ is the centroid of the ith category. Hence, *N* centroids are obtained in each task. To a query sample $x_{q}$ in *Q*, it is also transferred by DCT and embedded by $f_{\theta }$ to be a vector $V_{q}$. The probability that the sample $x_{q}$ belongs to category $C_{i}$ is decided by the distances from $V_{q}$ to the group of centroids:

(3) $$p\left(y=C_{i}|x_{q},S\right)=\frac{exp(<g\left(f_{\theta }\left(d\left(x_{q}\right)\right)\right),g\left(C_{i}\right)>)}{\sum_{i\in N}exp\left(<g\left(f_{\theta }\left(d\left(x_{q}\right)\right)\right),g\left(C_{i}\right)>\right)}$$

where *g* denotes the Gaussian-like calibration and <.,.> denotes the distance between two vectors. The objective is defined in Formula (4). In training, we use cross entropy loss to train our model:

(4) $$\theta \underset{}{\leftarrow }\underset{\theta }{argmin}-\frac{1}{N\times W}\sum_{x_{q}\in Q}^{}\sum_{c\in C}^{}y_{qc}log\left(P\left(y=c|x_{q},S\right)\right)$$

where θ represents the trainable parameters; *C* is the category set of *S*; $y_{qc}$ is a sign value, which equals 1 when $y_{q}=c$ and equals 0 when $y_{q}\neq c$, *y* is the prediction category; $P(y=c|x_{q},S)$ is the possibility of query sample $x_{q}$ belonging to class *c* and being supported by *S*.

#### 4.2.3. DCT Module

**Figure 8:**
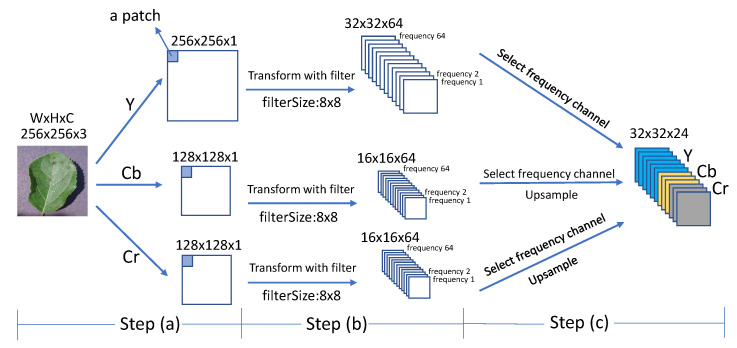
The pipeline of the DCT module. Step (a): The 2D RGB image is transformed to three channels: Y, Cb and Cr. Then, the channels are separated into patches as the size $S_{DCT}\times S_{DCT}$. Step (b): The Y, Cb and Cr channels are filtered by different frequent filters and reshaped to frequency cube. In the cube, each channel represents each frequency. Step (c): Informative frequency channels are selected to be frequency representations of the sample.

The processing flow in the DCT module is shown in Figure 8. First, all images are unified to the same size. In Step (a), referring to the JPEG compression method, the original RGB image is converted to a YCbCr image. *Y* represents brightness, Cb and Cr represent color. Since the human visual system is more sensitive to brightness than to color, we aim to retain more brightness information and follow the 4:2:0 chroma subsampling ratio [46,47]. For example, the RGB image is 256×256×3 after resizing. After converting to YCbCr, the *Y* channel is 256×256×1, and the Cb channel and Cr channel are both 128×128×1.

**Figure 9:**
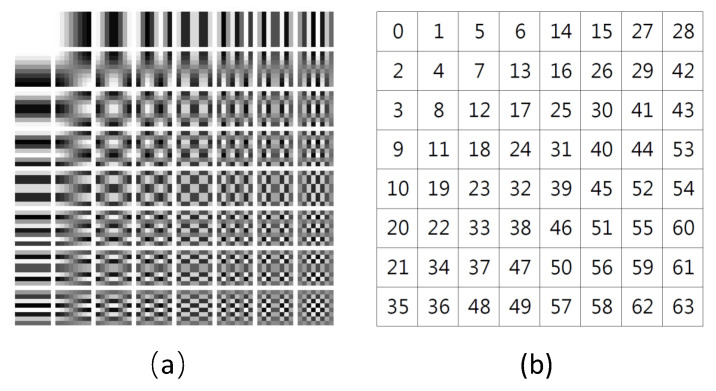
(**a**) The DCT basis images, forming the complete DCT dictionary of size 8×8. (**b**) The numbered basis images; the numbers are listed as the shape “Z” from 0 to 63.

In Step (b), each channel is divided into patches of size $S_{DCT}\times S_{DCT}$ (e.g., 8×8), the same size as the DCT filter:

(5) $$X=\left\{X_{pq}|p\in \left[0,\left\lfloor H/S_{dct}-1\right\rfloor \right],q\in \left[0,\left\lfloor W/S_{dct}-1\right\rfloor \right]\right\}$$

where $X_{pq}$ denotes each patch of channel *X* and (p,q) indicates the position of each patch. Then, the $S_{DCT}\times S_{DCT}$ DCT is performed on each patch. The 2D DCT for one patch can be expressed in matrix form as [48]:

(6) $$Z_{pq}=CX_{pq}C^{T}$$

where $Z_{pq}$ is the frequency representation of the patch $X_{pq}$ in the DCT domain, *C* denotes the DCT metric and $C^{T}$ is the transpose metric of *C*. *C* is calculated as:

(7) $$C_{i,j}=\left\{\begin{array}{cc} \frac{1}{\sqrt{S_{DCT}}} & i=0\\ \sqrt{\frac{2}{S_{DCT}}}cos\left(\frac{(2j+1)i\pi }{2S_{DCT}}\right) & i>0 \end{array}\right.$$

where $i,j\in (0,\ldots ,S_{DCT}-1)$. In the frequency representation of each patch, the lower frequencies are in the left-top corner and higher frequencies are in the right-bottom corner, which are illustrated in Figure 9. Figure 9a is a 8×8 2D DCT basis image, which shows the combination of horizontal and vertical frequencies [48]. Each step of movement from left to right yields a half-cycle increase in the horizontal frequency, and each step of movement from top to bottom yields a half-cycle increase in the vertical frequency. In Figure 9b, each digit in the matrix represents a frequency (8×8 filter yields 64 frequencies). These digits are listed as “Z” from left-top corner to right-bottom corner in order from low to high frequency. For example, in the *Y* channel, totally, $\left\lfloor H/S_{dct}-1\right\rfloor \times \left\lfloor W/S_{dct}-1\right\rfloor$ values of each frequency are obtained. In each channel (*Y*, Cb, Cr), these values are reshaped to a cube by grouping instances with the same frequency into a sub-channel. Each channel in a cube represents a certain frequency. The number of frequencies is decided by filter-size, which is $S_{dct}\times S_{dct}$ (e.g., 8×8).

In Step (c), the more impactful frequencies are selected from the three frequency cubes *Y*, Cb and Cr. Many researches indicate that not all frequencies are significant for a given task [28,29]. The less informative frequency channels can be removed without any performance degradation. By discarding the useless channels, the size of input data is reduced, along with the computational complexity and communication bandwidth. The meaningful frequencies (e.g., top-24 channels) are selected and concatenated together to be the final frequency representation of an image. The details of frequency selection are introduced in Section 4.2. Since the size of elements in Cb and Cr channels is half that of elements in the *Y* channel, it is necessary to upsample the elements of Cb and Cr so that they are the same size as the elements of *Y* before concatenation. The concatenated frequencies are used for the subsequent feature extraction by backbone network.

#### 4.2.4. Gaussian-like Calibrator

**Figure 10:**
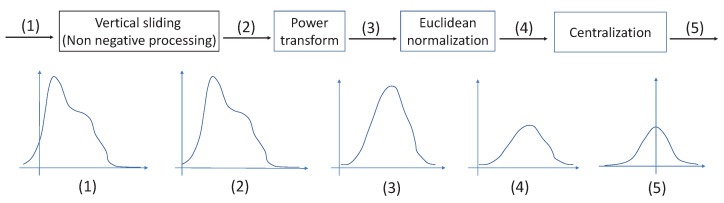
The schematic diagram of the GC module. (1) The histogram of the raw feature distribution, (2) The histogram after vertical sliding. (3) The histogram after power transform. (4) The histogram after normalization. (5) The histogram after centralization.

A Gaussian-like calibrator is used to calibrate the skewed features. The group of centroid vectors of support set *S* and the feature vectors of query set *Q* are fed into the GC module for calibration. The sequential processing of the calibration consists of four steps, as shown in Figure 10. The five curves represent the histograms of the inputs and outputs of the steps in this schematic diagram.

Before the PT, all values must be non-negative because the PT is calculated as the square root when the power value is equal to 0.5. However, the raw feature vectors may contain negative values. Hence, non-negative processing is needed before PT. The vertical sliding shifts the feature vector along the *y*-axis so that all values are non-negative without changing its distribution.

Tukey (1977) describes an orderly way of re-expressing variables using a power transformation [35], which is defined as:

(8) $$v^{\prime }=\left\{\begin{array}{cc} {\left(v+\epsilon \right)}^{\beta } & \;\mathrm{if}\;\beta >0\\ log(v+\epsilon ) & \;\mathrm{if}\;\beta =0\\ -({\left(v+\epsilon \right)}^{\beta }) & \;\mathrm{if}\;\beta <0 \end{array}\right.$$

where $v=\left[v_{1},\ldots ,v_{i},\ldots ,v_{d}\right]\in R^{d}$ is a *d*-dimensional vector, 1≤i≤d, $v_{i}$ denotes the value in the *i*th position, ϵ = 1 × 10^−6^. v+ϵ is used to ensure that the values of *v* are strictly positive. β is a hyper-parameter. The skewed distribution can be adjusted by changing β. Generally, if a distribution is right-skewed (positively skewed), decreasing β can phase out the right-skewed distribution, and phase into a left-skewed (negatively skewed) distribution when β becomes negative. Note that β=1 leads to no effect.

An euclidean normalization is often combined with PT, which is used to scale the features to the same area to avoid the large variance values predominating over the others [36]. The formula is defined as:

(9) $$v^{\prime }=\frac{v}{{∥v∥}_{2}}$$

At the end, the centralization makes all values symmetrical on the *y*-axis.

#### 4.2.5. Distance Measurement

After the Gaussian-like calibration, a set of high-dimensional calibrated vectors is ready for distance measurement. In meta-learning, the purpose is not to learn the specific knowledge of the training categories, but to learn “how to distinguish different categories”. Therefore, the classifier is no longer a traditional classifier, such as a linear classifiers, but is replaced by a distance measurement module.

A distance metric uses distance function which provides a relationship metric between each element of the input [49]. It is used to find the data pattern in order to make data-based decisions in many machine learning algorithms. In this work, cosine similarity is used to calculate the distance between the vector of a query sample and the centroid of the category, which is defined as Formula (10). It is a measure of similarity between two non-zero vectors of an inner product space and measured by the cosine of the angle between two vectors, to determine whether two vectors are pointing in roughly the same direction [50].

(10) $$\begin{array}{c} <A,B>=\frac{A\cdot B}{∥A∥∥B∥}=\frac{\sum_{i=1}^{d}A_{i}B_{i}}{\sqrt{\sum_{i=1}^{d}A_{i}^{2}}\sqrt{\sum_{i=1}^{d}B_{i}^{2}}} \end{array}$$

## 5. Conclusions

Given that most existing studies are in the spatial domain, we introduced the FSL of image-based plant-disease recognition into the frequency domain in this work. The DCT module and the GC module were proposed for improving the feature representations of samples and the generalization ability of the model. From the experimental results, we found that the CNNs can extract better features from frequency representation, and the Gaussian-like calibration is beneficial to the metric-based methods. The two modules are easily adapted to CNNs, and combining them achieves the best result. The settings of data in this work mainly simulated two application scenarios: mixed-plant target and single-plant target. For the first case, when extending a new category, the identification accuracy can reach 95% with a 5-shot,5-way task. To the latter one, when extending a new disease of a species, the accuracy can reach 80% with the 5-shot,5-way task. Even the increase in N-way could degrade the accuracy, increasing the number of shots that can offset the drawback. The results indicate that using FSL in an application of plant-disease recognition is worthwhile. In brief, we provided a new perspective for dealing with FSL in the frequency domain. Our method has wide universality, which can be used in other related tasks. In the frequency domain, there is huge potential for exploration.

## Figures and Tables

**Table 1:** The experimental results of the three data settings. ($f_{\theta }$: Resnet12; filter-size: 8 × 8; full channels: 192; s: spatial domain; f: frequency domain).

ID	Method	Channel	1-Shot	5-Shot	10-Shot	20-Shot	30-Shot	40-Shot	50-Shot
	setting-1								
e1	s	-	79.32	91.19	92.53	93.55	93.84	93.97	93.86
e2	s + GC	-	83.08	93.15	94.71	95.75	95.90	96.01	95.84
e3	f	top-24	84.99	94.92	96.71	95.91	97.15	97.31	97.62
e4	f + GC	top-24	**86.34**	**95.30**	**96.93**	**97.48**	**97.62**	**97.83**	**98.01**
	setting-2								
e5	s	-	78.27	90.96	92.69	93.60	93.76	94.02	94.03
e6	s + GC	-	79.81	92.11	93.58	94.26	94.51	94.50	94.74
e7	f	top-24	82.85	94.48	95.58	96.35	96.59	96.78	96.77
e8	f + GC	top-24	**85.41**	**95.21**	**96.17**	**96.66**	**96.90**	**97.04**	**97.15**
	setting-3								
e9	s	-	57.46	75.12	79.32	81.41	82.48	83.32	83.50
e10	s + GC	-	60.69	79.38	82.78	85.19	86.18	86.87	86.86
e11	f	top-24	62.32	80.37	83.57	85.75	86.51	**87.11**	87.22
e12	f + GC	top-24	**64.54**	**80.89**	**84.06**	**85.91**	**86.67**	87.01	**87.33**

The bolded values indicates the highest performance in each data setting.

**Table 2:** The results of different channel selection strategies. e13 uses the frequency combination for ImageNet; e7 uses the frequency combination for setting-2. ($f_{\theta }$: Resnet12; method: f; data: setting-2; filter-size: 8 × 8).

ID	Channel	Y-Cb-Cr	Y	Cb	Cr	1-Shot	5-Shot	10-Shot
e13	top-24	14-5-5	0, 1, 2, 3, 4, 5, 6, 7, 8, 9, 10, 14, 15, 20	0, 1, 2, 6, 9	0, 1, 2, 6, 9	79.66	93.56	95.24
e7	top-24	11-7-6	0, 1, 3, 4, 5, 8, 12, 16, 24, 32, 40	0, 2, 7, 8, 10, 17, 27	0, 1, 3, 7, 8, 16	**82.85**	**94.48**	**95.58**

The bolded values indicates the highest performance in this table.

**Table 3:** The results of different top-N frequencies selected from full 192 channels. ($f_{\theta }$: Resnet12; method: f; data: setting-2; filter-size: 8 × 8).

ID	Channel	Y-Cb-Cr	Y	Cb	Cr	1-Shot	5-Shot	10-Shot
e14	top-8	4-2-2	0, 8, 24, 40	0, 7	0, 16	80.53	93.08	94.39
e15	top-16	8-4-4	0, 1, 4, 5, 8, 24, 32, 40	0, 7, 8, 17	0, 7, 8, 16	81.34	93.73	95.05
e7	top-24	11-7-6	0, 1, 3, 4, 5, 8, 12, 16, 24, 32, 40	0, 2, 7, 8, 10, 17, 27	0, 1, 3, 7, 8, 16	**82.85**	94.48	95.58
e16	top-32	14-8-10	0, 1, 2, 3, 4, 5, 8, 12, 16, 24, 32, 34, 35, 40	0, 2, 7, 8, 10, 16, 17, 27	0, 1, 2, 3, 7, 8, 16, 17, 24, 32	82.25	94.10	95.36
e17	top-48	25-9-14	0, 1, 2, 3, 4, 5, 6, 8, 12, 16, 20, 24, 32, 34, 35, 36, 39, 40, 43, 49, 51, 56, 58, 59, 60	0, 2, 7, 8, 10, 16, 17, 18, 27	0, 1, 2, 3, 5, 7, 8, 10, 16, 17, 18, 19, 24, 32	81.84	93.71	94.94
e18	top-64	38-11-15	0, 1, 2, 3, 4, 5, 6, 7, 8, 12, 16, 20, 22, 24, 31, 32, 34, 35, 36, 38, 39, 40, 41, 43, 45, 46, 48, 49, 51, 53, 54, 55, 56, 58, 59, 60, 61, 62	0, 2, 7, 8, 10, 16, 17, 18, 19, 26, 27	0, 1, 2, 3, 4, 5, 7, 8, 10, 16, 17, 18, 19, 24, 32	82.84	**94.49**	**95.77**
e19	64(Y)	64-0-0	0-63	-	-	71.01	86.61	88.71

The bolded values indicates the highest performance in this table.

**Table 4:** The results of top-24 channels selected from different ranges. ($f_{\theta }$: Resnet12; method: f; data: setting-2; filter-size: 8×8).

ID	Channel	Y-Cb-Cr	Y	Cb	Cr	1-Shot	5-Shot	10-Shot
e7	top-24/192	11-7-6	0, 1, 3, 4, 5, 8, 12, 16, 24, 32, 40	0, 2, 7, 8, 10, 17, 27	0, 1, 3, 7, 8, 16	**82.85**	**94.48**	**95.58**
e20	top-24/108	11-6-7	0, 1, 3, 4, 5, 8, 12, 16, 20, 24, 32	0, 7, 8, 10, 16, 27	0, 1, 2, 7, 8, 16, 24	80.20	92.50	94.24
e21	top-24/84	11-6-7	0, 1, 2, 3, 4, 5, 8, 12, 16, 20, 24	0, 7, 8, 16, 19, 27	0, 1, 2, 7, 8, 16, 24	81.23	93.79	95.19

The bolded values indicates the highest performance in this table.

**Table 5:** The results of different filter-sizes and top-N frequency selections. The full channels of filter-size 4×4, 8×8 and 16×16 were 48, 192 and 768, respectively. ($f_{\theta }$: Resnet12; method: f; data: setting-2).

ID	Channel	Y-Cb-Cr	Y	Cb	Cr	1-Shot	5-Shot	10-Shot
filter-size: 4×4								
e22	top-6	3-2-1	0, 3, 8	0, 8	0	81.15	93.42	94.87
e23	top-12	5-3-4	0, 1, 2, 3, 8	0, 2, 8	0, 3, 5, 8	80.65	92.67	94.26
e24	full-48	16-16-16	0-15	0-15	0-15	80.84	92.59	94.12
filter-size: 8×8								
e7	top-24	11-7-6	0, 1, 3, 4, 5, 8, 12, 16, 24, 32, 40	0, 2, 7, 8, 10, 17, 27	0, 1, 3, 7, 8, 16	**82.85**	**94.48**	**95.58**
e25	top-48	25-9-14	0, 1, 2, 3, 4, 5, 6, 8, 12, 16, 20, 24, 32, 34, 35, 36, 39, 40, 43, 49, 51, 56, 58, 59, 60	0, 2, 7, 8, 10, 16, 17, 18, 27	0, 1, 2, 3, 5, 7, 8, 10, 16, 17, 18, 19, 24, 32	81.84	93.71	94.94
e26	full-192	64-64-64	0-63	0-63	0-63	81.62	93.55	95.04
filter-size: 16×16								
e27	top-96	45-26-25	0, 1, 2, 3, 4, 17, 19, 32, 34, 37, 40, 43, 52, 54, 56, 58, 71, 73, 82, 85, 96, 103, 106, 107, 113, 117, 142, 144, 145, 150, 158, 170, 173, 187, 188, 190, 200, 201, 208, 219, 240, 242, 244, 253, 254	0, 1, 2, 17, 23, 46, 69, 75, 76, 90, 126, 129, 131, 140, 160, 164, 193, 196, 200, 207, 216, 217, 224, 229, 231, 252	0, 33, 46, 51, 54, 59, 71, 74, 85, 87, 100, 111, 112, 164, 166, 169, 172, 189, 199, 234, 237, 238, 249, 252, 254	80.93	92.84	94.42
e28	top-192	82-50-60	0, 1, 2, 3, 4, 13, 17, 19, 25, 27, 28, 29, 32, 34, 37, 38, 40, 41, 43, 46, 52, 53, 54, 56, 58, 60, 62, 71, 73, 82, 85, 94, 96, 99, 103, 106, 107, 113, 117, 122, 126, 141, 142, 144, 145, 150, 154, 158, 161, 168, 170, 173, 174, 179, 180, 184, 187, 188, 190, 192, 200, 201, 206, 208, 209, 211, 212, 218, 219, 223, 224, 240, 242, 244, 252, 253, 254, 255	0, 1, 2, 17, 23, 36, 45, 46, 58, 66, 69, 75, 76, 79, 90, 92, 97, 112, 115, 117, 119, 126, 129, 131, 132, 140, 143, 147, 157, 160, 161, 164, 165, 169, 180, 193, 196, 200, 205, 207, 210, 212, 216, 217, 219, 224, 229, 231, 235, 238, 240, 252	0, 7, 17, 18, 26, 32, 33, 34, 36, 39, 45, 46, 48, 51, 52, 53, 54, 59, 60, 71, 74, 82, 85, 87, 90, 98, 99, 100, 103, 111, 112, 135, 153, 154, 156, 158, 164, 166, 169, 172, 173, 177, 181, 183, 185, 189, 196, 198, 199, 210, 219, 229, 234, 237, 238, 245, 246, 249, 250, 252, 254	81.73	93.63	95.05
e29	full-768	256-256-256	0-255	0-255	0-255	81.47	93.41	94.87

The bolded values indicates the highest performance in this table.

**Table 6:** The ablation experiments of the GC module. ($f_{\theta }$: Resnet12; data: setting-2; method: f + GC; filter-size: 8×8; channel: top-24).

ID	Components of GC Module	1-Shot	5-Shot	10-Shot
e30	PT	81.82	93.27	94.91
e31	PT + normalization	82.60	94.55	95.95
e32	PT + centralization	85.36	94.94	96.08
e8	PT + normalization + centralization	**85.41**	**95.21**	**96.17**
e33	normalization + PT + centralization	85.04	94.76	95.92

The bolded values indicates the highest performance in this table.

**Table 7:** The results of different values of β. ($f_{\theta }$: Resnet12, data: setting-2, method: f + GC, filter-size: 8×8, channel: top-24).

ID	β	1-Shot	5-Shot	10-Shot
e34	1.5	83.13	93.57	94.77
e35	1	84.74	94.82	95.88
e8	0.5	**85.41**	**95.21**	96.17
e36	0	84.75	95.08	**96.37**
e37	−0.5	69.44	78.84	79.73
e38	−1	68.10	77.19	78.22

The bolded values indicates the highest performance in this table.

**Table 8:** The results of integrating the spatial and frequency representations. ($f_{\theta }$: Resnet12; data: setting-2; filter-size: 8×8; channel: top-24).

ID	Method	1-Shot	5-Shot	10-Shot
e6	s + GC	79.81	92.11	93.58
e8	f + GC	**85.36**	**94.86**	**96.13**
e39	s + f + GC	84.08	94.35	95.92

The bolded values indicates the highest performance in this table.

**Table 9:** The results compared with related works.

		Data Setting and K-Shot					
ID	Method	1-Shot	5-Shot	10-Shot	Y	Cb	Cr
	Data setting in [10]						
	Siamese Contrastive [10]	50.2	64.2	70.2	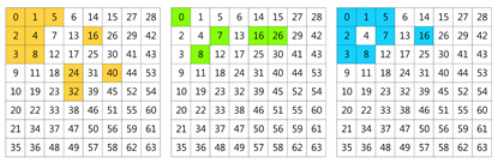		
	Siamese Triplet [10]	65.2	72.3	76.8			
	Single SS [13]	74.5	89.7	92.6			
	Iterative SS [13]	75.1	90.0	92.7			
	MAML [40,41]	58.8	79.3	87.1			
	IMAL [40]	63.8	83.5	89.9			
e40	Ours s	76.4	91.0	93.2			
e41	Ours s + GC	78.7	91.0	92.3			
e42	Ours f	79.8	92.5	94.3			
e43	**Ours f + GC**	**81.6**	**93.1**	**94.3**			
	Split-1 of [13]						
	Single SS [13]	33.7	50.9	66.7	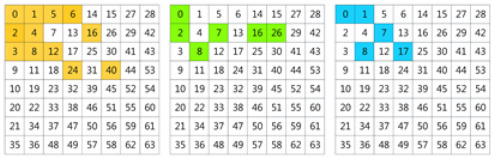		
	Iterative SS [13]	34.0	53.1	68.8			
	[19]	46.6	63.5	-			
e9	Ours s	57.5	75.1	79.3			
e10	Ours s + GC	60.7	79.4	82.8			
e11	Ours f	62.3	80.4	83.6			
e12	**Ours f + GC**	**64.5**	**80.9**	**84.1**			
	Split-2 of [13]						
	Single SS [13]	44.7	74.7	85.7	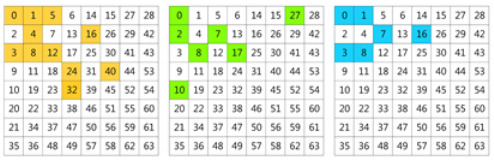		
	Iterative SS [13]	46.4	76.9	89.2			
	[19]	70.9	87.0	-			
e5	Ours s	78.3	91.0	92.7			
e6	Ours s + GC	79.8	92.1	93.6			
e7	Ours f	82.9	94.5	95.6			
e8	**Ours f + GC**	**85.4**	**94.9**	**96.1**			
	Split-3 of [13]						
	Single SS [13]	52.3	67.6	79.9	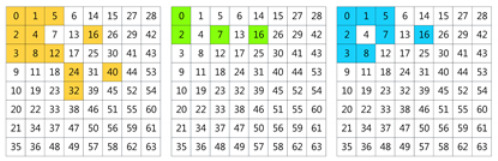		
	Iterative SS [13]	55.2	69.3	80.8			
	[19]	75.4	88.5	-			
e44	Ours s	76.6	88.6	90.7			
e45	Ours s + GC	81.2	90.0	91.5			
e46	Ours f	79.9	90.8	92.7			
e47	**Ours f + GC**	**82.7**	**92.1**	**93.7**			

The bolded values indicates the highest performance in the comparable group.

**Table 10:** The three data settings of PV used in our work.

Data Setting	Source Set (28)	Target Set (10)	Target Species
setting-1	2, 3, 4, 5, 7, 9, 10, 11, 13, 14, 15, 18, 20, 22, 23, 24, 25, 26, 28, 30, 31, 32, 33, 34, 35, 36, 37, 38	1, 6, 8, 12, 16, 17, 19, 21, 27, 29	apple, cherry, corn, grape, peach, pepper, potato, strawberry, tomato, orange
setting-2	5, 8, 9, 10, 11, 16, 17, 18, 19, 20, 21, 22, 23, 24, 25, 26, 27, 28, 29, 30, 31, 32, 33, 34, 35, 36, 37, 38	1, 2, 3, 4, 6, 7, 12, 13, 14, 15	apple, grape, cherry
setting-3	1, 2, 3, 4, 5, 6, 7, 8, 9, 10, 11, 12, 13, 14, 15, 16, 17, 18, 19, 20, 21, 22, 23, 24, 25, 26, 27, 28	29, 30, 31, 32, 33, 34, 35, 36, 37, 38	tomato

## Data Availability

Data are available from its original source cited in the article. The PlantVillage dataset is available at https://data.mendeley.com/datasets/tywbtsjrjv/1 (accessed on 20 July 2022).

## Abbreviations

The following abbreviations are used in this manuscript:

FSL	few-shot learning
PV	PlantVillage (dataset)
CNN	convolutional neural network
GC	Gaussian-like calibration
DCT	discrete cosine transform
PT	power transform
SE	Squeeze-and-Excitation (attention module)
